# Deep Learning Models for the Diagnosis and Screening of COVID-19: A Systematic Review

**DOI:** 10.1007/s42979-022-01326-3

**Published:** 2022-07-25

**Authors:** Shah Siddiqui, Murshedul Arifeen, Adrian Hopgood, Alice Good, Alexander Gegov, Elias Hossain, Wahidur Rahman, Shazzad Hossain, Sabila Al Jannat, Rezowan Ferdous, Shamsul Masum

**Affiliations:** 1 Faculty of Technology, The University of Portsmouth (UoP), Portland Building, Portland Street, Portsmouth, PO1 3AH UK; 2 Time Research and Innovation (TRI), 189 Foundry Lane, Southampton, SO15 3JZ UK; 3 336/7, TV Road East Rampura, Khilgaon, Dhaka 1219 Bangladesh; 4 School of Computing, University of Portsmouth (UoP), Lion Terrace, Portsmouth, PO1 3HE UK

**Keywords:** Coronavirus (COVID-19), RT-PCR, Machine learning (ML), Deep learning (DL), X-ray images, Computed tomography (CT) images

## Abstract

COVID-19, caused by SARS-CoV-2, has been declared as a global pandemic by WHO. Early diagnosis of COVID-19 patients may reduce the impact of coronavirus using modern computational methods like deep learning. Various deep learning models based on CT and chest X-ray images are studied and compared in this study as an alternative solution to reverse transcription-polymerase chain reactions. This study consists of three stages: planning, conduction, and analysis/reporting. In the conduction stage, inclusion and exclusion criteria are applied to the literature searching and identification. Then, we have implemented quality assessment rules, where over 75 scored articles in the literature were included. Finally, in the analysis/reporting stage, all the papers are reviewed and analysed. After the quality assessment of the individual papers, this study adopted 57 articles for the systematic literature review. From these reviews, the critical analysis of each paper, including the represented matrix for the model evaluation, existing contributions, and motivation, has been tracked with suitable illustrations. We have also interpreted several insights of each paper with appropriate annotation. Further, a set of comparisons has been enumerated with suitable discussion. Convolutional neural networks are the most commonly used deep learning architecture for COVID-19 disease classification and identification from X-ray and CT images. Various prior studies did not include data from a hospital setting nor did they consider data preprocessing before training a deep learning model.

## Introduction

SARS-CoV-2, known as coronavirus, causes COVID-19. It is an infectious disease first discovered in China in December 2019. In a short period of time, it became a global pandemic [1]. The rate of transmission of this virus is very high, as it spreads from human to human through droplets, while talking or sneezing. The survival rate of the virus itself is high, and it lives from a few hours to a few days in the environment [2]. Older people with an existing illness are most likely to suffer from it, and symptoms (cough, fever, shortness of breath or difficulty breathing and running nose) are only visible in 40–45% of individuals [3]. Nonetheless, there are also a considerable number of individuals with multiple conditions aged between 20 and 51, who have suffered (severe respiratory illness or pneumonia) and died from the disease [4–6].

The gold-standard method for testing for coronavirus is the RT-PCR (reverse transcription-polymerase chain reaction). It is highly sensitive, specific, reliable, widely accepted, and the average testing time to conclude a result is 6–8 h [7]. Therefore, if a patient tests positive for coronavirus after testing their sample through this method, then the result is close to 100% accurate and efficient. The second consideration is the sensitivity, which denotes the ability of RT-PCR test to eliminate any types of false-negative results. Here, false negatives can be defined as failing to identify the virus when the virus is present in the patient’s sample. Since RT-PCR shows poor sensitivity, if a patient tests negative through RT-PCR then it is not certain whether he or she is actually COVID-19 negative or not [8–10].

Machine learning (ML) is widely used for medical diagnosis to identify diseases in some domains, and it plays a significant role in diagnosing and screening critical conditions [11, 12]. AI and ML are used to identify potential causes and symptoms of COVID-19 from chest radiography or chest computed tomography (CT) scan images [11]. However, at the beginning of this global pandemic, scientists, researchers, doctors, and healthcare professionals from different parts of the world were designing and modelling new ML or deep learning (DL) techniques for early diagnosis and screening of this disease. It has become difficult to choose an accurate and appropriate model for real-life applications from the theoretical proposals as there has been insufficient research. We have observed that a lot of models have low accuracy and other performance metrics compared with the gold standard that is RT-PCR. Furthermore, the RT-PCR testing kits are not easily accessible in low- and middle-income countries [13]. Thus, researchers and scientists are trying to find alternative solutions all over the world. The 90-min test has been rolled-out across NHS hospitals in the United Kingdom. It is a fast and quick test compared to RT-PCR, and it can distinguish the COVID-19 virus from winter flu viruses that have similar symptoms. Scientists who have developed this system hope that it will help to reduce the spread of coronavirus in the community [3, 14]. This article deals with a DL-based diagnosis or screening method for COVID-19 patients, so we have excluded the 90-min test from our analysis.

It is a matter of concern that there are underlying factors like the lack of experienced personnel and adequate equipment to consider in the diagnosis and screening of COVID-19 using deep learning algorithms. The limitations can only be overcome when all the problems are identified and understood. This study has described and indicated the novel and previous approaches that have been utilised in the case of COVID-19 image classification. We have critically analysed each article and extracted the main contributions along with the limitations of the proposed deep learning models in terms of their performance for diagnosis and screening of COVID-19. Two popular imaging techniques have been addressed along with some additional techniques, where X-ray and CT images play a substantial role in the diagnosis of this virus. This research has highlighted the image classification methods, various datasets, diagnosis techniques, image data partitioning methods and the accuracy of various DL algorithms. Furthermore, this research has also identified some of the factors that lead to low scores in image medical classification and lack of robustness in the case of COVID-19 screening and testing. This review will serve as a benchmark and will play a significant role for future researchers to undertake related DL research and increase their performance.

This study presents a systematic review to demonstrate the analysis and performance of recently proposed methods based on DL to diagnose COVID-19. It will help readers to understand machine-learning models and their applicability in real life. Each section and subsection will present different aspects of the study. In “Methodology”, we discussed the research question, search and selection strategy, quality assessment, and other necessary processes that helped us organise and conduct the research. “Results and Discussion” discusses the systematic search results and observations, leading to “Summary of COVID-19 Diagnosis Using Deep Learning and Conclusion”.

## Methodology

**Fig. 1:**
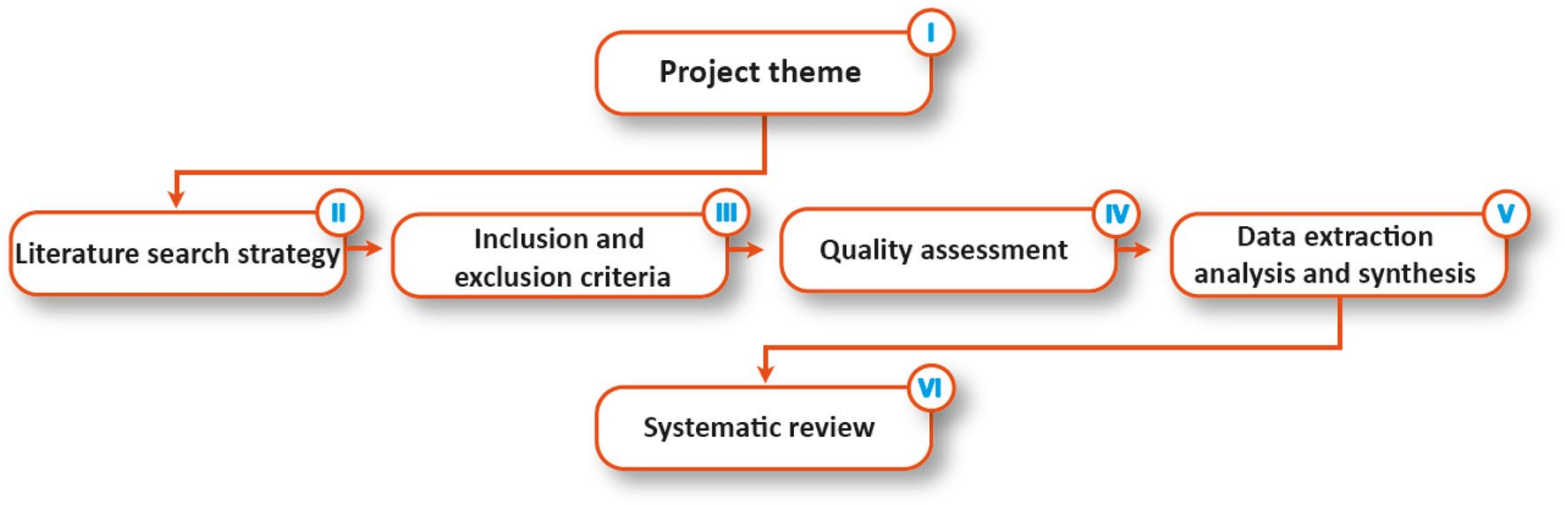
Overall project methodology

The project methodologies of this study are based on the research questions and the project theme, as shown in Fig. 1. The objectives and the project settings influenced the systematic review methodology to design the study that consisted of three stages: planning, conduction, and analysis/reporting. In the conduction stage, the literature is searched and articles are identified or selected based on the inclusion and exclusion criteria. In the analysis/reporting stage, all the selected papers are reviewed and analysed.

## Research Question

There are diverse and growing numbers of novel methods based on many DL frameworks to identify or classify COVID-19 patients and research studies based on AI. However, our investigation focuses on whether the DL models from this large number of research studies are practically suitable for diagnosing COVID-19. Therefore, to understand the underlying problem, we have set a research question: “How effective are the present proposed artificial intelligence and machine-learning models of many researchers for the diagnosis and screening of COVID-19?”.

### Search and Selection Strategy

**Fig. 2:**
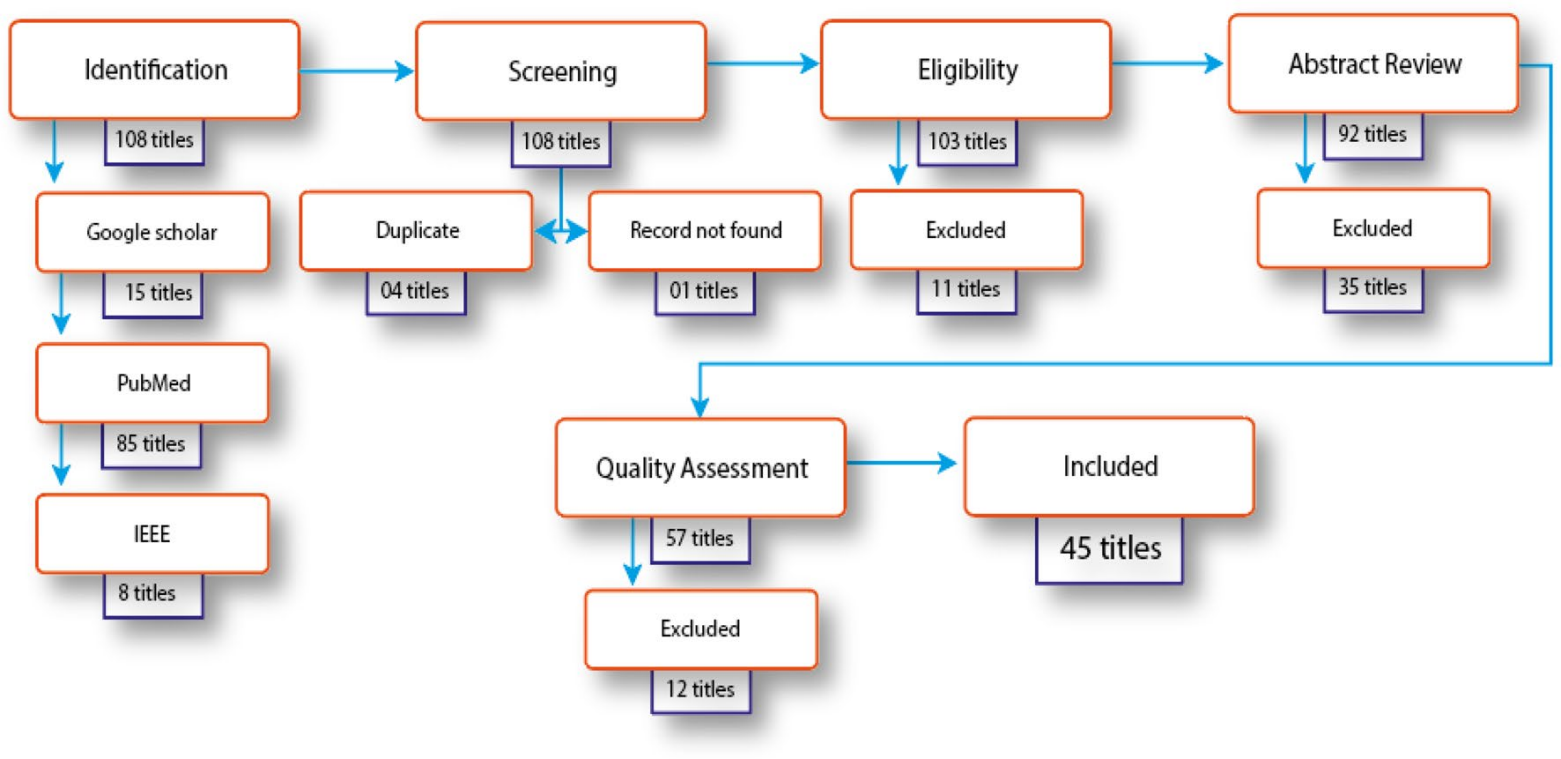
Systematic literature search and selection flowchart

We have searched various electronic databases from December 2019 to September 2020, as shown in Fig. 2. We have used Google Scholar, PubMed, and IEEE to identify relevant published papers, as they are well established and include a wide range of peer-reviewed up-to-date journals, information, and reports. A systematic literature search strategy was developed by following “A structured approach to documenting a search strategy for publication” [15] framework. We have used three search terms (deep learning, screening, COVID-19) and Boolean operators like 'and' between the search keywords during the search process. The keywords are identified based on the research question we have set. After the initial selection, every paper is then checked against the quality assessment tool to qualify for the review.

### Selection Criteria

We have set our selection criteria for accepting a research article based on the following key criteria and retrieved only those that meet these conditions.

#### Inclusion Criteria

- The research paper must be a journal article or conference paper.
- The research study must contain a DL-based model or framework designed solely for COVID-19 purposes.
- The purpose of each research study must be focussed on the diagnosis or screening of COVID-19 patients through DL methods.
- The time frame for each research study is December 2019 to December 2021.
- The presented AI and ML models must be effective for the diagnosis and screening of COVID-19.

#### Exclusion Criteria

- Any research work that is published as a preprint, early works or not peer-reviewed.
- Any comparative studies.
- Editorials, review papers or research letters.

### Quality Assessment

For quality assessment of this study, we have followed the NIH “Quality Assessment of Systematic Reviews and Meta-Analyses” and “The methodological quality assessment tools for preclinical and clinical studies, systematic review and meta-analysis, and clinical practice guideline: a systematic review” [16]. This work has chosen four researchers to perform the quality assessment on the selected papers, and we have followed [17] for an effective result. In the quality assessment stage, the researchers assessed the selected articles using a 10-point rating scale and answering ten individual questions, as shown in Table 1. The threshold value was defined as 75 and they rated the articles according to the predefined questions. If the paper scored greater than or equal to 75, the papers have been included for review; otherwise, the paper has been discarded.

**Table 1:** Quality assessment questionnaire for this systematic review [16, 18]

No.	Questions	Yes	No	Other	Score
1	Is the review based on a focussed question that is adequately formulated and described?				
2	Were eligibility criteria for included and excluded studies predefined and specified?				
3	Did the literature search strategy use a comprehensive, systematic approach?				
4	Were titles, abstracts, and full-text articles dually and independently reviewed for inclusion and exclusion to minimise bias?				
5	Was the quality of each included study rated independently by two or more reviewers using a standard method to appraise its internal validity?				
6	Were the included studies listed along with essential characteristics and results of each study?				
7	Was the publication bias assessed?				
8	Was heterogeneity assessed? (This question applies only to meta-analyses.)				
9	Were the primary data collected and stored?				
10	How consistent is the information obtained from one source with information available from other sources?				

Quality Rating	Good	Fair	Poor	Total score	
Rater 1 Initials					
Rater 2 Initials					

## Results and Discussion

This section will discuss the data extraction and our observations based on the literature. Thus, this section is divided into “Systematic Search Results”, “Observation” and “Discussion”.

### Systematic Search Results

In the initial primary research, 108 titles were retrieved and collected for the title review, comprising 15 titles from Google Scholar, 85 titles from PubMed and 8 titles from the IEEE. At the next stage, all the retrieved titles were checked for duplication and availability, and a total of four titles were deleted for duplication and one article for availability. After an initial skip through, a total of 103 papers were downloaded for review. The papers were then checked according to exclusion criteria. 11 papers were excluded, and 92 papers were selected for abstract review. During the abstract review, 35 articles were found not to propose new DL models so, after excluding these 35 articles, a total of 57 papers were included for full-text review. Following assessment of these 57 articles using the quality assessment rules, 12 papers failed the quality assessment criteria. Therefore, finally, we have included 45 papers for the full review.

### Observation

**Fig. 3:**
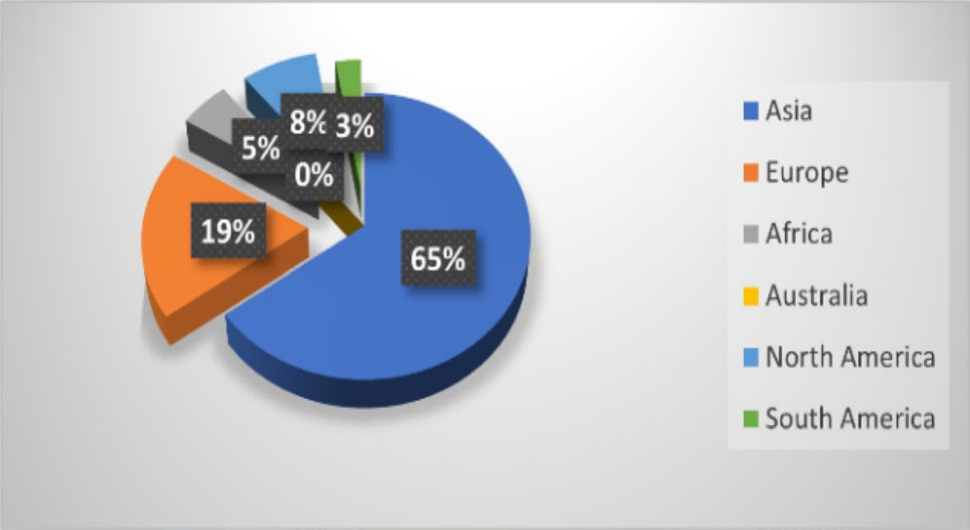
COVID-19 research around the globe

A DL model is designed to automate the diagnostic process of COVID-19 patients. Thus, for this study, we have to consider its effectiveness and importance at the beginning. It is observed that although Europe and USA are leading developing life-saving drugs [19], AI and DL research is worldwide. This study tried to find appropriate articles based on the inclusion and exclusion criteria from different continents and subcontinents to build effective models. Figure 3 illustrates the number of papers we have taken to complete our systematic review. Europe is the second and has the majority of papers compared to Africa, North America, and South America. No articles from Australia qualified for our assessment.

**Fig. 4:**
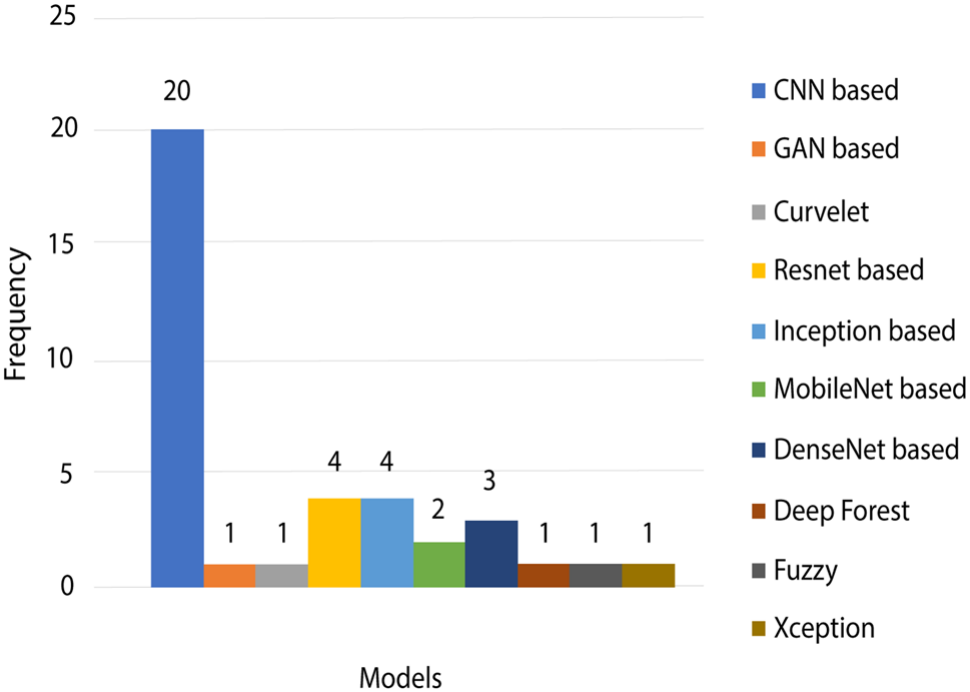
Deep learning models for detecting the COVID-19 patients

Furthermore, we have also observed the DL model used in various research articles for classifying or detecting COVID-19 patients. Figure 4 illustrates the number of models used in multiple settings, where convolutional neural network (CNN)-based DL models are the most popular. Second are the ResNet and inception network models. There are other models such as fuzzy approaches, deep forest, GAN based or curvelet-based methods, but they are not used widely for detecting COVID-19.

**Fig. 5:**
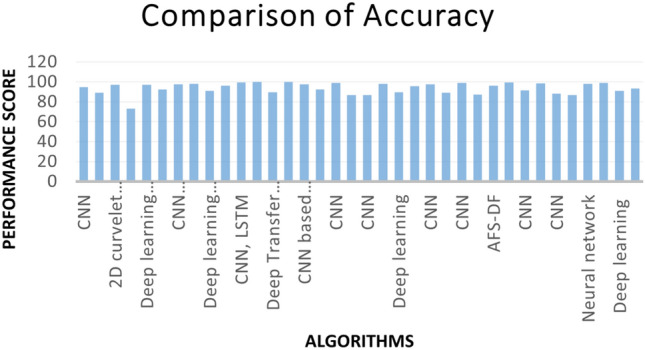
Accuracy of the models and the study

We have also observed the accuracy of each paper shown in Fig. 5. Most of the work has been carried out on the CNN algorithm, and various CNN architectures have been used in previous studies. In addition to these, bidirectional algorithms (BI-LSTM), deep transfer learning, and VG16 have also been applied in an earlier study. By looking at the graph, it can be observed that all the algorithms’ performance scores are close. It also observed some gaps in some of the studies; for example, some researchers did not significantly change the data preprocessing. Some worked on a small amount of data, which affected the performance score. Since ML or DL approaches require large datasets to predict a satisfactory result, it was also observed that some of the results were very good with a small dataset, especially for medical image analysis. We have also observed that the input or training datasets used in various DL models are a major concern. The image selection and measurement process in multiple studies are ambiguous, and it is not clear whether the chest X-ray or CT images are collected effectively and reliably.

It is also not clear that the predicted outputs of the DL models and the outcomes are applicable in real life. The authors of the various studies have only trained and tested their models in a lab environment and did not include a real-life implementation. Many studies did not consider data preprocessing before training a DL model. Therefore, it is not clear how these models will perform with the real dataset. Due to noise or image blurring, the clinical images generated from various medical devices are sometimes inaccurate and contain several defects and errors. Therefore, it is necessary to consider data preprocessing since the ML or DL models are prone to bias introduced by missing or noisy data [20].

### Discussion

In this section, we will describe every model included in the review. We have divided it into three distinct subsections: models based on X-ray imaging, CT images, and other models. We will describe them first and then compare the contributions and express our observations.

#### Model-Based on X-Ray Imaging

##### Deep Neural Network [21]

These models are an alternative RT-PCR method based on chest X-ray images and deep learning. It is designed for the automated diagnostic purpose of COVID-19 patients and comprises an end-to-end architecture. The diagnosis is obtained through raw chest X-ray images. The authors used 125 chest X-ray images to train the proposed deep learning model. The diagnosis is also performed after 5–13 days of the already recovered patients. However, in this model, the authors did not consider any feature extraction methods from the input images and as well as did not consider any preprocessing of the raw images. Since raw medical images may contain defects or error values that the deep learning models cannot eliminate; instead, the deep learning models may bias these error values.

##### Patch-Based Deep Learning [22]

The authors propose a patch-based deep neural network model, which can be trained on a small or limited dataset but with high performance. The authors first investigated various imaging features of CXR, such as lung area intensity distribution and cardiothoracic ratio. The investigation demonstrates significant differences among the patch wise intensity distribution. Therefore, based on these findings, a novel patch-based deep neural network is proposed, and final classification results are obtained through majority voting from inference results. Since the patch training is included, network complexity has been significantly reduced. Therefore, the neural network can be trained efficiently and avoid overfitting with the limited dataset. The authors considered preprocessing highly recommended for better training the deep models.

##### CovXNet [9]

A deep convolutional neural network is called CovXNet. It can effectively extract chest X-rays’ features by varying depth-wise convolution and changing dilation rates. Since the dataset of COVID-19 patients is small, transfer learning with the ImageNet dataset is adopted to train the proposed deep learning models. First, a dataset with ordinary pneumonia and COVID-19 X-ray samples are used for transfer learning. Then, the proposed deep learning model named CovXNet is used to detect COVID-19 from the X-ray images. Second, the trained convolution layers are transferred directly through fine-tuning layers for training on smaller COVID-19 X-rays. Sensitivity is an essential feature for measuring deep learning models’ performance in medical practice [12]; the authors did not consider the sensitivity in their experiment.

##### CNN and LSTM Based [23]

In this model, a deep CNN and LSTM network is combined to diagnose COVID-19 patients at an early stage automatically. A dataset of chest X-ray images is formed containing 4575 images. A deep learning-based system has been depicted, combining CNN and LSTM network to detect the COVID-19 from the chest X-ray images automatically. The authors used CNN as a feature extraction technique to extract the features. Based on these extracted features, LSTM network is used to classify the COVID-19 patients. Since the LSTM network has internal memory, it can learn from long-term states. In LSTM, directed graph is used to connect the nodes. Thus, according to the study, 2D CNN and LSTM network improve classification accuracy. The sample size is minimal, which needs to be enhanced to increase the model’s generalisability. A changing dilation rate has been proposed based on varying depth-wise convolution to extract the features from chest X-rays effectively.

##### Fuzzy Colour and Stacking [24]

To accelerate the detection of COVID-19 symptoms and prevent further spreading, the authors have proposed this model. Since deep learning-based methods or computer-aided tools for diagnosing and screening COVID-19 patients are accurate and take less time to test. Therefore, it can prevent the spreading of the virus by identifying or classifying positive patients from others. Through preprocessing, various features are extracted from the input image data. In addition, the stacking technique is introduced, which superimposes each pixel from equivalent images. The authors used SMO algorithm to extract features. Then the feature set is combined to enhance the classification performance of the proposed model. MobileNet-v2 and SqueezeNet are combined to perform the classification tasks. The authors used preprocessing steps in the input images since the medical images are pronto miss values and include noise. Pre-processing can help mitigate these erroneous data and improve system performance.

##### Decision Tree Classifier [25]

A deep learning-based decision tree classifier is proposed to classify the COVID, infected patients, from regular patients. The decision tree classifier is divided into three parts. The authors used PyTorch frame-based convolutional neural network to train their model. It also includes three steps binary decision tree classifier. The radiographers first evaluate the CXR images, and then only the good quality images are taken as the input of the deep learning-based prediction model. The model predicts TB, COVID-19 and other diseases. If the detection result is for COVID-19, then the physician’s assessment is added for further diagnosis. Here, data preprocessing are not considered, and the radiologists are involved in measuring the quality of the images, which may lead to a human error.

##### VGG and CNN Based [26]

The authors proposed a deep learning system to detect lung diseases from X-ray images by joining VGG, information increase, and spatial transformer organisation (STN) with CNN. This new mixture strategy is named VGG Data STN with CNN (VDSNet). As execution instruments, Jupyter Scratchpad, TensorFlow, and Keras are utilised. Although every research is conducted based on some real-life problem, this research has some drawbacks which can be responsible for many issues. The authors claimed that their approach is quite useful than other traditional algorithms; however, they experimented with a small dataset, showing good accuracy.

##### Combine Deep Models [27]

This model is proposed to fight against COVID-19 pneumonia. Since deep learning algorithms have been utilised in medical and health research, the author claimed that it is crucial to implement an automatic detection and classification system as a speedy elective finding choice to forestall COVID-19 spreading among individuals. This paper leads a comparative study of utilising the novel deep learning models (VGG16, VGG19, DenseNet201, Inception ResNet V2, Inception V-3, ResNet50, and MobileNet V2) to manage the discovery and characterisation of COVID-19 pneumonia. This study has various drawbacks; for instance, the advanced features extraction technique is missing. This work is impressive, but it may not be suitable when it is considered for production level since the accuracy expresses that this study requires more datasets for training and improving the model performance.

##### Explainable Deep Learning [28]

The research provides a fully automated and quick diagnosis system with deep learning. This study aims to detect the disease and COVID-19 from X-ray images by applying the deep learning algorithm. This paper proposes a threefold approach aimed at: (i) detecting whether a chest X-ray is associated with a stable patient or a patient with the generic pulmonary disease; (ii) distinguishing between generic pulmonary diseases and COVID-19 and, once COVID-19 has been identified, (iii) highlighting the symptomatic areas of COVID-19 in the chest X-ray. This study considers a deep learning network based on the VGG16 model by leveraging transfer learning to create the first and second models. Although this study achieved an excellent score to fight against the difficulties, this proposed study cannot evaluate the proposed method with a more comprehensive pulmonary disease set. This method will work well on small data, but the larger data size will be needed when applied in real life.

##### nCOVnet [29]

This research proposed a deep learning neural network-based approach, “nCOVnet” an alternative fast screening procedure that can be used to identify the COVID-19. A dataset consisting of about 192 X-ray images of COVID-19-positive patients and 337 images was gathered from this research. This model relies on the deep learning algorithm called convolutional neural network (CNN). This algorithm consists of 24 layers. convolution + ReLU and Max Pooling layers have been combined in this research. Since the X-ray images belong to a different size, the dataset has been converted into 224 × 224 pixels. These layers are part of the pre-trained model VGG16 and are trained in the dataset ImageNet. ImageNet comprises approximately 15 million annotated images from 22,000 several groups, and 92.7% accuracy on ImageNet was achieved by VGG16. Although the authors achieved good accuracy, several limitations can be found in this study. The author did not compare their study with the published research, such as LSTM.

##### Flat and Hierarchical [30]

In this study, the authors proposed an effective solution for identifying COVID-19 in flat and hierarchical Chest X-ray images scenarios for classification. This research suggested a classification schema considering the multiclass classification and hierarchical classification since pneumonia can be found to be structured as a hierarchy. Using some well-known texture descriptors and a pre-trained CNN model, the classification scheme extracts characteristics. The proposed model achieved F1-Score 0.65 using a multiclass method and F1-Score 0.89 for the hierarchical classification recognition of COVID-19 cases. This research has some limitations. This method will work well on small data, but more extensive data will be needed when applied in real life. It might face difficulties on accuracy with the real and big data set. Furthermore, the deficiency of the cross-validation approach might not achieve the overall objective.

##### CNN Based [31]

This paper proposed an infected tissue diagnosis and identification of COVID-19 patients based on lung X-ray imaging using coevolutionary neural network approaches (CNN). This study has been considered three deep learning algorithms to diagnose COVID-19 patients using X-ray images of the lung. The authors used two deep learning algorithms two identify the phenomenon, to illustrate, deep neural network (DNN) and convolution neural network (CNN). The approaches have been directly utilising for lung disease. The comprehensive study showed that CNN architecture performs with better accuracy (93.2%). Several drawbacks can be found in this proposed system: (i) this research did not consider any types of model validation approaches like ROC-AUC. It could impact the model when applied to real-life; (ii) the dataset amount is not much longer, so it could impact while detecting the original sample.

##### COVIDiagnosis-Net [32]

In this study, the authors have developed a deep Bayes-SqueezeNet-based diagnosis of the coronavirus disease 2019 (COVID-19) from X-ray images. This study has proposed an AI-based effective solution for detecting COVID-19, COVID diagnosis-Net, based on deep SqueezeNet with Bayes optimisation. The author claims that tuning the parameter is exceptionally significant to obtain better accuracy. To overcome the imbalance issues of the proposed public dataset, this study performs a multiscale augmentation process. The CNN architecture has been considered, and the overall accuracy is 0.983. This research introduces a new model for the Rapid Diagnosis of COVID-19 centred on the COVIDiagnosis-Net, called deep Bayes-SqueezeNet. In this analysis, a multiscale offline increase is performed to address the public dataset’s imbalance issue.

##### Convolutional Capsnet [33]

The authors have proposed their system by utilising convolutional capsnet. The new network architecture has two classes: binary class and multiclass carried out by capsule network. The COVID-19 and other images have been resized to be the capsule network’s input to work correctly with the proposed scheme. They have utilised X-ray images for COVID-19, no-findings and pneumonia to build the dataset. They have also suggested the architecture of a new network for the classification of COVID-19. They have used an augmentation process to optimise the preprocessing of data. Several drawbacks have been noticed during the study of the paper. First, the authors only worked with X-ray images. Second, the authors trained their model with a limited dataset. As the classification of COVID-19 disease is a vital issue, this model might give a better performance if it is trained with a larger dataset.

##### GAN Based [34]

The paper’s authors had utilised GAN-based radiographs (CXR) augmentation to evaluate the performance and diagnosis of chest-related illness, such as COVID-19 chest radiographs. They also developed the multiclass deep learning classification patterns to diagnose abnormalities in chest X-ray scans. The authors adopted deep learning GAN-based synthetic data to train their proposed model. To prepare their dataset, the authors had utilised radiographic changes in CT images and GAN-based synthetic data. Four significant types of deep learning architectures, including deep learning mechanism with image augmentation, transfer learning using Inception V-3, ResNet architecture without image augmentation, and classification with target classes, were enriched. Several limitations have been identified from this study. Initially, the authors had utilised X-ray images from the frontal view, but no lateral and rare view had not been enumerated.

##### 2D Curvelet Transformation Algorithm [35]

In this study, the authors illustrated an interconnected combined architecture with meta-heuristic optimisation, two-dimensional curvelet transformation algorithm, and deep learning mechanism to investigate the patients affected with coronavirus from X-ray images. The authors had applied 2D curvelet transformation to images obtained from a patient chest X-ray images and interpreted a feature matrix utilising obtained coefficients. Later, the coefficients anticipated from the feature matrix are enhanced with the chaotic slap swarm algorithm (CSSA). They have also applied a deep learning paradigm to diagnose COVID-19 patients utilising EfficientNet-B0 architecture. We have observed that the proposed methodology is quite impressive, and the authors have seen good results in their experiment. If the authors combined the X-ray images and symptoms, COVID-19 patients, the result would be uncountable.

##### Pruned Deep Learning Ensembles [36]

The authors had illustrated a model using an ensemble of iteratively pruned deep learning models for investigating COVID-19 pneumonia with chest X-ray images from bacterial pneumonia and generals. The authors had utilised a convolutional neural network (CNN) and a selection of ImageNet pre-trained architecture for training and evaluating at the patient level on chest X-ray images. The scheme was then taught modality-specific features. Later, the system was transferred and fine-tuned to optimise performance and classify chest XR as bacterial, showing ordinary or COVID-19 pneumonia. We have observed that their primary concern was the dataset size; because of the data size and inherent variability, additional computational resources are required for effective establishment and utilisation. The result will be uncountable if the system is trained with a large dataset and highly configured computer devices. The authors considered only X-ray images for classifying the COVID-19; however, they did not focus on computed tomography images to diagnose COVID-19.

##### Bayesian Optimisation [37]

The authors have aimed to develop an effective CNN with a rich filter family to detect COVID-19. The authors had implemented a reliable scheme using a deep neural architecture to assist the radiologists, specialists and physicians. A cheap and fast detection methodology also had been proposed in this manuscript. The proposed scheme was based on the CNN architecture and investigated some discriminative features on chest X-ray images. The proposed CNN architecture had the following characteristics: weight-sharing abstraction and convolution with rich filters and trained from scratch. The authors utilised a unique serial network in five convolutional layers instead of using a pre-trained CNNs model. The CNN architecture used a feature extractor of deep neural networks. Later, the authors had applied machine learning such as k-nearest neighbour and support vector machine upon extracted features. We have observed that the authors used a novel detection method of COVID-19 patients. The authors used five layers of CNN architecture instead of using a pre-trained model on chest X-ray images, which indicates a significant contribution to the diagnosis of infected patients. The study is quite impressive.

##### CoroNet [38]

The paper’s authors had presented a deep learning approach based on CNN to diagnose COVID-19 infection from X-ray images. The proposed model is based on deep CNN architecture to segment three types of pneumonia such as normal, bacterial and COVID-19. They had also introduced binary and three-class variants of their theoretical model and compared the outcomes with other literature studies. However, the proposed method can be a possible solution for COVID-19 patients’ diagnoses. The authors used a small dataset for the model training, and the model shows good performance on a small dataset. However, this model is scalable, which means this model can achieve high accuracy on a larger dataset and require less computational resources.

This study interprets the summary of each article that has worked on COVID-19 diagnosis utilising X-ray images. Table 2 shows the corresponding table for the short overview of the models. This table illustrates the adopted models, dataset size, accuracy and contribution of the papers reviewed in this section.

**Table 2:** Summary of each article that worked with X-ray images on COVID-19 diagnosis

References	Adopted models	Dataset size	Accuracy (%)	Contribution
[34]	DNN	80,000	89.00	Optimisation of deep learning architecture and its hyperparameters to improve the performance of the model
[35]	EfficientNet-B0	2905	99.69	A combined model consisting of two-dimensional (2D) curvelet transformation, meta-heuristic optimisation algorithm and deep learning techniques are proposed here
[26]	Hybrid CNN	4143	73.00	Developed a new hybrid algorithm suitable for predicting lung disease from X-ray images
[28]	VGG	6523	97.00	Proposed approach represents a suggestion for the radiologist to immediately localise the necessary X-ray areas
[27]	NA	5856	92.00	A comparison between different deep convolutional neural network (DCNN) algorithms to automatically classify the X-ray images
[31]	DNN, CNN	682	93.20	Presented two AI-based methods for classification and diagnosis of patients and normal people lung MRI images
[23]	CNN-LSTM	4575	99.40	Developed a combined deep CNN-LSTM network to automatically assist the early diagnosis and presented a detailed experimental analysis
[38]	CoroNet	1300	89.60	Proposed DCNN model to classify three different types of pneumonia, bacterial pneumonia, viral pneumonia and COVID-19 pneumonia
[9]	CovXNet	5856	97.40	An efficient scheme is proposed for training DNN so that the trained parameters can be effectively utilised
[37]	CNN	2905	98.97	Provided a cheap, fast, and reliable intelligence tool for COVID-19 infection detection
[21]	CNN, ResNet50	147	99.30	The proposed approach has proved to be highly effective for various medical imaging applications
[29]	nCOVnet	337	97.00	Presented a fast detection method using X-ray image analysis and obtained results evaluated by three different parameters
[30]	NA	1144	89.00	Identification of different types of pneumonia caused by multiple pathogens using only CXR images
[36]	CNN	179	99.01	Highlighted the benefits due to the use of an ensemble of iteratively pruned DL models
[24]	MobileNetV2, Squeeze Net	295	99.27	Provides 100% success rate in detecting disease by examining the X-ray images of COVID-19 patients, minimises the interference in every image in the dataset and provides efficient features with stacking techniques
[33]	CapsNet	231	97.24	A novel network architecture has been introduced in the study
[32]	COVIDiagnosis-Net	1203	98.30	Presents a novel model for the rapid diagnostic and overcomes the imbalance problem of the public dataset
[25]	ResNet18	585	98.00	Investigates the feasibility of using deep learning-based decision tree classifiers for detection of COVID-19 from CXR images

**Fig. 6:**
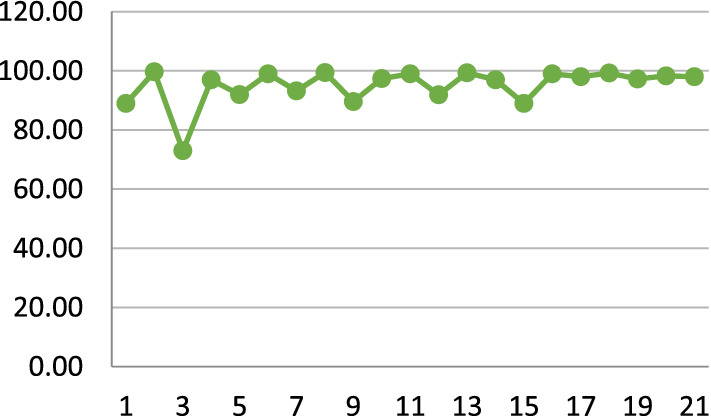
Variation in highest accuracy of the models

**Fig. 7:**
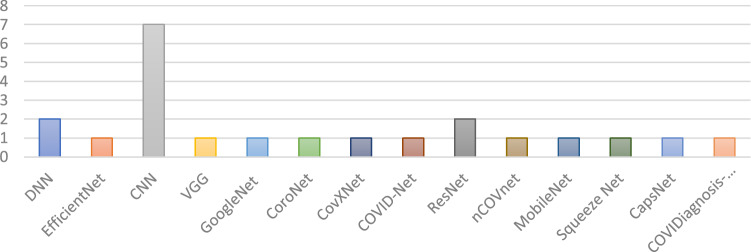
The occurrence of models that worked on X-ray images

Albahli [34] worked with the largest number of samples using a DNN model. Almost all the published models achieved an accuracy of more than or close to 90%, except for the model proposed by Bharati et al. [26], as shown in Fig. 6. This research faced some challenges while handling the large-scale dataset, so the accuracy rate is low compared with the other proposed works. Figure 7 shows the occurrence of different models applied to X-ray images.

#### Model Based on CT Images

##### Deep Forest [39]

Using chest CT images, an adaptive feature selection guided deep forest model has been proposed for COVID-19 classification. First, the location-specific features are extracted from the CT images. Then, the deep forest model is introduced to learn these high-level features from the extracted images. This mechanism enhances the performance of the model. Feature selection and classifier are integrated into a unified framework. They have used the trained model for COVID-19 prediction. The experimental result shows that the proposed model can achieve a superior result with chest CT images. The accuracy, sensitivity is above 90%, and specificity is nearly 90%.

##### DenseNet201 [1]

The proposed deep transfer learning model is based on DenseNet 201; hear a deep transfer learning approach is proposed for classifying the patients as COVID infected or not. Transfer learning is suggested due to the scarcity of training data. The model includes the convolution layer, max-pooling layer, average pooling layer, global average pooling layer, dense block, and softmax layer. The filter size used is 7 * 7 or 1 * 1 with stride size of 2. The dataset contains 2492 set of CT scan images. Among them, 1262 images are positive, and the rest of them are harmful. They have used transfer learning; however, transfer learning in case of clinical images is not suitable for real-life implementation.

##### MODE [40]

The authors implemented a scheme using CNN, ANN, and ANFIS models to classify affected patients. These models are applied on chest CT images to ensure good performance. The proposed system utilised feature extraction, multi-objective fitness function, and multi-objective differential evolution, including mutation operation, crossover operation, and section operation. We have observed some limitations in this study. First, COVID patients’ classification from chest CT images is a big deal as alarming of patients worldwide. Second, the authors had proposed their model with one of the MODE-based CNN model mechanisms by considering specificity and sensitivity. Parameters tuning is needed to ensure more optimum results. Third, a deep neural network requires a more effective dataset to maintain better accuracy. Finally, the authors had not proposed any connectivity and RT-PCR, the most significant COVID-19 patient classification methodology.

##### Ni et al. [41]

They have aimed to develop a scheme that automatically detects and quantitatively evaluate the pneumonia lesions throughout the chest computed tomography (CT) of patients diagnosed with COVID-19. To work correctly with the proposed model, the authors had used two types of images; one is pneumonia Concussions CT images, and another is no abnormality on CT images. The proposed algorithm is performed in three interconnected parts. Concussions of CT images, segmentation and location, were trained and validated in the patients with chest CT images and established pathogen diagnosis. Here, quantitative model detection performance was compared to three radiological residents with two experienced radiologists reading as a reference norm by evaluating the precision, sensitivity, specificity and F1-score. While studying the paper thoroughly, we have noticed several observations. The authors work with a large dataset of CT images. They have performed their research work at three hospitals during the Corona outbreak in China. The proposed architecture can subsidise COVID-19 patients to diagnose coronavirus disease.

##### Machine-Agnostics Segmentation [42]

The authors had presented a novel strategy of confirming COVID-19 using modern computation. They have proposed a quantification and fast, precise and machine-agnostics segmentation method for CT-based diagnosis of COVID-19. The authors had utilised computed tomography-based images to analyses the classification of COVID-19 patients. The authors had performed the augmentation to enrich the dataset. After that, the authors have first taken a CT scan simulator of real coronavirus patients by fitting the dynamic changes of data measured at a particular time point. After that, a novel deep learning algorithm which decomposes 3D segmentation strategy into three 2D ones. We have identified several drawbacks in this study. First, the authors need to establish an organised platform to perform their research. Federated learning can be a possible solution to overcome this issue. Second, the model should be trained with a large dataset to optimise accuracy. Finally, the authors need to maintain orthogonal information sources for the model, like epidemiology. Thus, the ambiguous cases of COVID-19 patients can be eliminated, and a better diagnosis will be achieved.

##### Multidimensional Dataset [43]

This study has developed an artificial intelligence (AI) algorithm on chest CT utilising information from a worldwide assorted, multinational dataset. The authors demonstrate and claim that robust models can accomplish up to 90% exactness in free test populaces, keeping up high particularity in non-COVID-19 related pneumonia, and exhibiting adequate generalizability to concealed patient populaces/focuses. This study proposed two approaches for separating COVID-19 from other clinical substances. To illustrate, the entire 3D model has experimented with CT’s trimmed lung area to a predefined size (192 × 192 × 64 voxels) for input to the research algorithm. Hybrid CT resampled the cut lung side of CT to deified exploration (1 mm × 1 mm × 5 mm) and sampled multiple 3D regions (192 × 192 × 32) for input to the research algorithm. In this study, various drawbacks can be found because it is commonly said that no system is 100% accurate if it is an AI-based solution. First, model preparation was restricted to patients with positive RT-PCR testing and coronavirus-related pneumonia on chest CT to separate between COVID-19-related ailment and different pathologies. Second, the authors claimed that they developed an AI solution on Chest CT, but despite the neutral RT-PCR experiment, CT is often negative.

##### Inf-Net [44]

In this model, a novel COVID-19 lung infection segmentation deep network (Inf-Net) is proposed to consequently recognise contaminated regions from chest CT slices. In this investigation, an equal incomplete decoder is utilised to total the significant level highlights and produce a worldwide map. At that point, the understood opposite consideration and express edge consideration are used to demonstrate the limits and upgrade the portrayals. The authors utilised a semi-supervised segmentation architecture dependent on a haphazardly chosen propagation technique, which requires a couple of labelled centres pictures and uses unlabeled data. The authors claimed that their approach could enhance the learning capability and achieve the best accuracy. In this type of benchmark, several drawbacks and limitations are identified. The authors conduct a two-step methodology to accomplish multiclass disease naming, leading to imperfect learning execution.

##### Zhang et al. [45]

Numerous COVID-19 patients infected by SARS-CoV-2 infection create pneumonia (called novel COVID pneumonia, NCP) and quickly progress to respiratory disappointment. In any case, fast analysis and recognisable proof of high-risk patients for early intercession are tested. Based on this purpose, this study has been proposed as a practical solution, and this is the motivation and key idea of this study. The author of this study provided an AI-based effective solution to combat the prognosis of COVID-19 pneumonia. The AI solution can diagnose NCP and separate it from other common diseases. The radiologist, as well as physician, will be benefiting from this AI system. On the other hand, the authors claimed that their proposed solution could identify significant clinical markers. This is the principal contribution of this proposed study.

##### Wang et al. [46]

In this research, to support COVID-19 diagnostic and predictive analysis, the authors proposed a novel fully automatic DL system using a raw chest CT image (computed tomography). They used a two-step transfer learning strategy to automatically let the DL system mine lung features without requiring any time-consuming human annotation. First, with CT imaging and EGFR gene sequencing, this research obtained 4106 lung cancer patients. The DL system has learned hierarchical lung characteristics through training in this broad CT-EGFR dataset and can also represent them. Despite the excellent performance of the DL system, this analysis has many drawbacks. First, predictive cases, like death or admission to an intensive care unit, have not been considered in this review. Second, the treatment of extreme and moderate COVID-19 is different. Therefore, it would be useful to independently investigate the prognosis of COVID-19 in these two classes.

##### 2D Deep Learning Framework [47]

The authors claimed that CV images showed the rapid development of AI technology to diagnose and differentiate COVID-19 pneumonia from non-COVID-19 pneumonia and non-pneumonia diseases. This study has proposed an AI solution to combat the outbreak. This study suggested a single 2D Deep learning framework: The First track COVID-19 network (FCONet) to diagnose COVID-19 pneumonia based on the chest CT Image. Using one of four state-of-the-art pre-trained deep learning models (VGG16, ResNet50, Inception-v3, or Xception) as a backbone, FCONet was built through transfer learning. Although this study achieved good accuracy, some limitations and drawbacks can be found in this type of particular benchmark. This research mainly used a split research data set; AI models have been validated. Therefore, the study was derived from the same sources as the data from the training determined. This may raise problems with generalizability and overfitting of the proposed model.

##### Xu et al. [48]

This study proposed a deep learning system to screen novel coronavirus disease 2019 pneumonia. In this analysis, multiple CNN models were used to classify CT images datasets and measure the likelihood of COVID-19 infection. Such results could significantly assist in the early screening of COVID-19 patients. The overall performance rate was 86.7%. The deep learning model can diagnose accurately and screening COVID-19 patients, and this approach achieved good accuracy for frontline clinical doctors. Several limitations can be found in this study. First, the number of the training sample is not much more extensive; second, the number of training and test accuracy is not expanded to constitute some difficulties while applying it to real life.

##### Ouyang et al. [49]

The authors of this study developed a dual-sampling attention network for diagnosis of COVID-19 from community-acquired pneumonia. The authors claim that this approach could be vital to fighting against the COVID-19 outbreak. To automatically diagnose COVID-19 from community-acquired pneumonia (CAP) in chest computed tomography (CT). In particular, this study has proposed a new 3D coevolutionary network (CNN) online attention module to focus on infection regions in the lungs while making diagnostic decisions. This study has separated data from various hospitals, achieving a 0.944 AUC, 87.5% accuracy, 86.9% sensitivity, 90.1% precision, and 82.0% F1-score. Although this study achieved its objective, however, it has some drawbacks that did not solve. The proposed model used a 3D CNN network, but the accuracy is not satisfactory.

##### Matsuyama et al. [50]

Here, the authors utilised CNN- and ResNet-50-based model for distinguishing COVID-19 from non-COVID-19 cases. The input images to the CNN model are kept without any cropping and used the wavelet co efficient of entire images. To ensure that the CNN-based model is working correctly, the authors introduced a new model that is gradient weighted class activation mapping which produce a heat map. ResNet50 was pre-trained and transfer learning was adopted. From the results, it is clear that the CNN-based model can learn the extracted features well enough and successfully differentiate COVID-19 cases from non COVID-19 cases.

**Fig. 8:**
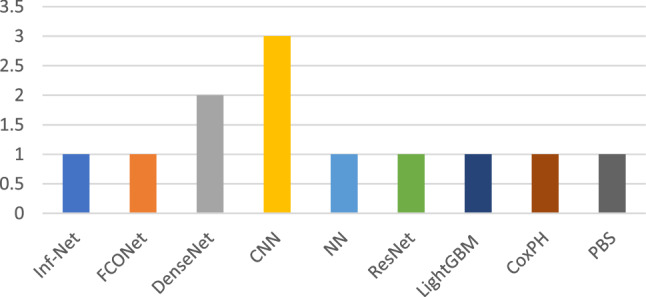
The occurrence of models that worked on CT images

**Fig. 9:**
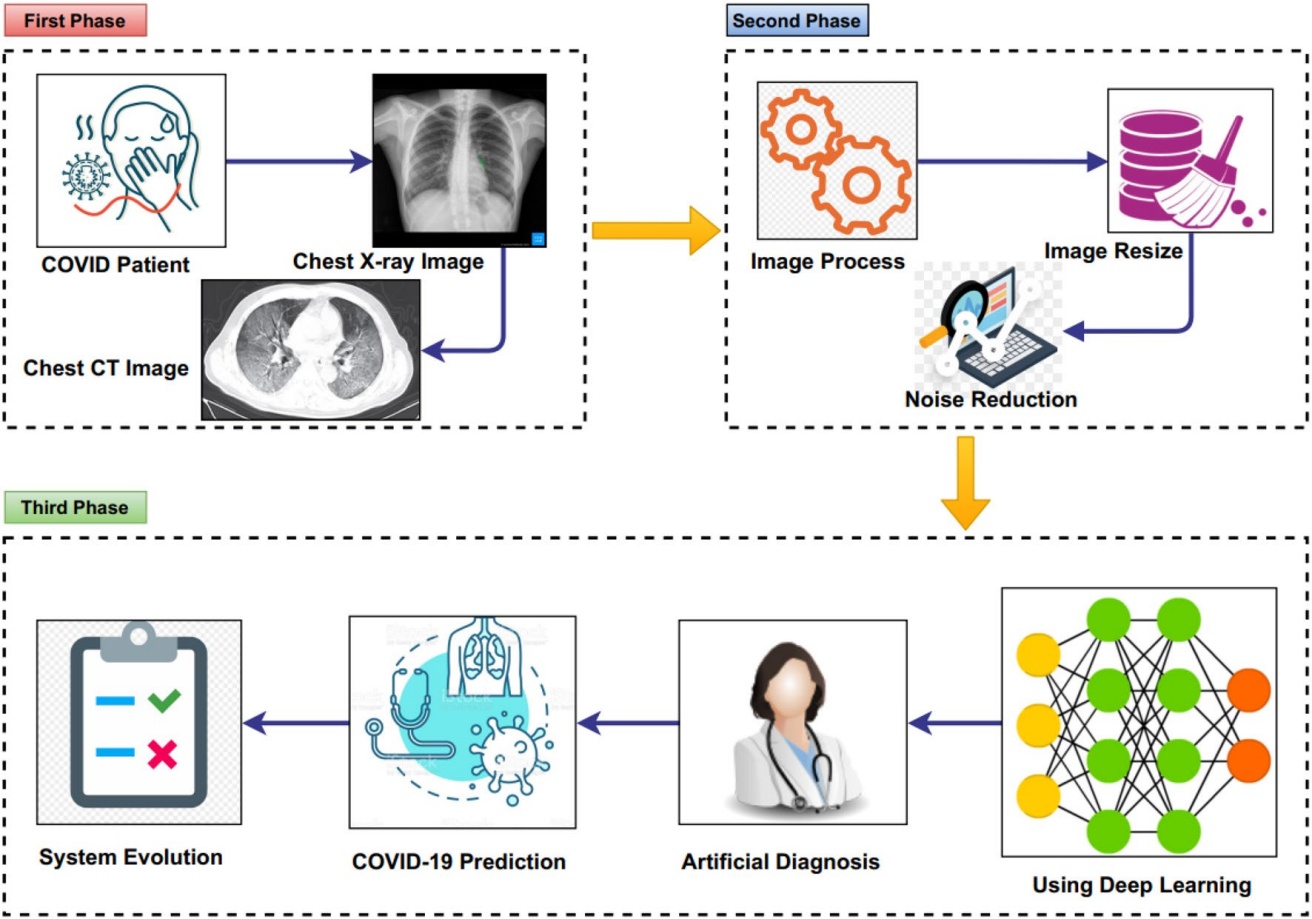
Diagnosis of COVID-19 using deep learning

This study presents the summary of articles that work on the COVID-19 diagnosis utilising CT images. Table 3 shows a short overview of the models. This table demonstrates the adopted models, dataset size, accuracy and contribution of the papers reviewed in this section. From Fig. 8, it can be seen that the most significant model proposed in this section is convolutional neural network (CNN). The second most proposed approach is based on the DenseNet model. The proposed work in paper [47] has achieved the highest accuracy using the FCONet model. On the other hand, the dataset considered here also includes a large dataset having 3993 samples. Among all of the proposed works in this section, the paper [48] has the lowest accuracy of 86.7%. The reason behind such low accuracy is the smaller number of model samples in the dataset in this study. Figure 9 shows the screening process of the COVID-19 diagnosis with deep learning.

**Table 3:** Summary of each article that worked with CT images on COVID-19 diagnosis

References	Model	Data size	Accuracy (%)	Contribution
[44]	Inf-Net	NA	NA	A novel COVID-19 lung infection segmentation deep network (Inf-Net) for CT scans has been presented
[43]	NA	2274	93.0	Presented robust models achieving up to 90% accuracy in independent test population and maintained high specificity in non-COVID-19-related pneumonia
[47]	FCONet	3993	99.9	Developed an AI technique using all available CT images from their own institution and publicly available data
[1]	DenseNet201	2492	97.0	Extensive experiments are performed to evaluate the performance of the proposed model on chest CT scans
[50]	CNN	720	92.2	Proposed method provided a promised computerised toolkit to assist radiologists serving as a second eye to classify COVID-19 from CT scans
[41]	UNet	96	NA	Higher sensitivity in detecting COVID-19 pneumonia was found compared with radiological alternatives and improved diagnosis efficiency by shortening processing time
[49]	CNN	2186	87.5	Proposed a dual-sampling strategy to train the network which alleviates the imbalanced distribution of the sizes of pneumonia infection regions
[40]	CNN	NA	2.0	A novel deep learning model is proposed using multi-objective differential evolution (MODE) and CNN for classification of COVID-19 patients
[39]	NN	2522	96.4	A novel adaptive feature selection guided deep forest method has been proposed for high-level deep features with a small number of medical image data for the classification between COVID-19 and CAP
[46]	DenseNet121-FPN	5372	NA	Provides a convenient tool for fast screening COVID-19 and finding potential high-risk patients assisting in medical resource optimisation and early prevention
[48]	ResNet18	618	86.7	Effective approach for the early screening of COVID-19 patients using deep learning model and proposed supplementary diagnostic method for assisting clinical doctors
[45]	LightGBM, CoxPH	4154	92.5	Proposed AI system can provide accurate clinical prognosis assisting clinicians to combat COVID-19
[42]	PBS	NA	90.0	Paper not relevant

#### Other Models

##### Abdani et al. [51]

This study proposed an effective solution to fight against the COVID-19 outbreak. The authors developed a Lightweight deep learning model to identify the COVID-19 disease. The authors claim that the lightweight model is significant since it can be deployed on various platforms, including a mobile phone and tablet. This model will only utilise a small portion of phone memory storage and indirectly contributes to a faster screening process. The proposed model is related to 14 deep convolutional neural network layers with an updated spatial pyramid pooling module. The multiscale capability of the proposed network enables COVID-19 disease to be recognised at different levels of severity. Based on the performance results, the proposed SPP-COVID-Net achieves the best mean precision of 0.946 with the lowest standard deviation between the training folds’ accuracy. It comprises a total number of parameters of only 862,331, using less than 4 megabytes. Even though this study achieved good accuracy, several drawbacks can be found in this study. First, the author did not reduce the dimension of the proposed research and proper scaling. They considered K-fold cross-validation, but ROC-AUC is missing, and they could have described it. In that way, we could have understood how the proposed model performs well in this type of work.

##### Han et al. [52]

The author of this study provided an AI-based effective solution to combat the prognosis of COVID-19 pneumonia. The AI solution can diagnose NCP and separate it from other common diseases. The radiologist, as well as physician, will be benefiting from this AI system. On the other hand, the authors claimed that their proposed solution could identify significant clinical markers. This is the principal contribution of this proposed study. There were two models in the NCP diagnostic system: a lung-lesion segmentation model and a diagnostic prediction model. First, this study has been trained by a segmentation network of 4,695 NCP and typical pneumonia patients with manually segmented images. The diagnosis classifier took the previous lung-lesion map as an input and created three classes of probability: NCP, common pneumonia, and normal classification network controls. Various prospective pilot studies have also been carried out to assess the performative AI. The author has stated that this study has improved accuracy by 92.49%. Although this study addresses its research question adequately, several drawbacks are found. First, the AI solution has been trained by a segmentation network of 4,695 NCP and typical pneumonia patients with manually segmented images. The system may not predict correctly for various disease-infected patients' inclusion since their accuracy is not good enough for this type of benchmark. Second, the amount of NCP and common pneumonia patients is much less in comparison.

##### Jiang et al. [53]

The paper’s contributions are classified into three significant parts. First, the authors merged the face recognition mechanism technology with dual-mode imaging to track out a data extraction respiratory scheme to detect wearing masks. The data required for the dual-camera algorithm were achieved from thermal videos of facial masks. Second, the authors proposed a classification scheme to investigate abnormal respiratory utilising a deep learning paradigm. In the end, the authors had developed a non-contact and competent health monitoring model using the data collected from the hospital. The authors had claimed that the proposed architecture could classify COVID-19 patients. While studying the manuscript, we have found two limitations. First is the measured distance from the camera module. The authors had claimed that their proposed method can detect respiration from 0 to 2 m. But, when the distance came up to 1.8 m, the proposed algorithm failed to analyse because of the gradually increased space. Second, one is the rotation difficulties of the camera during data measurement. The authors had defined the angle of rotation from 0° to 45°. The proposed method worked effectively within this range. But the proposed method was unavailable to measure data in pitch rotation.

##### Panwars et al. [29]

The authors of this study proposed an AI-based solution for the fast detection of coronavirus disease. They offered a deep transfer learning algorithm that speeds up identifying COVID-19 cases using chest X-ray and CT scan images. The proposed deep transfer learning algorithm focuses primarily on binary image classification to distinguish the different images for rapid and reliable COVID-19 identification, CXR, and CT scan images. The suggested algorithm considers the pre-trained weight to extract essential characteristics and learn the pattern of the COVID-19 sequence cases acquired from the CXR and CT scan images. The proposed approach’s key feature is entirely connected layers in the VGG19 model with five additional layers. CXR and CT scan images were considered here, including the cases of COVID-19, non-COVID-19, pneumonia, and regular. The proposed model achieved good accuracy with 95.61%. This study utilised the Grad-CAM technique that has several drawbacks. If the image includes several instances of the same class, Grad-CAM fails to localise objects in an image properly. This is a severe problem since a widespread phenomenon in the real world is multiple occurrences of the same object in a picture. The author did not describe how they have handled this issue.

##### Sedik et al. [54]

The contribution of this study is deploying machine, and deep learning models for efficient data-augmented detection of COVID-19 Infections. In that way, COVID-19 infections can be easily found through the efficient data-augmented approach. This study illustrates two data-argumentation models to enhance the learning capability of the novel deep learning algorithm, namely convolutional neural network (CNN) and the convolutional long short-term memory (ConvLSTM)-based deep learning models (DADLMs) and, this way, help the precision of COVID-19 discovery. The author proposed data-argumentation models to detect COVID-19 infections. This study has been utilised in various deep learning algorithms for solving the research problem. The author claimed that deep learning algorithms would play a vital role in this type of study to identify COVID-19 disease, and the objective of this study has been achieved.

##### Yoo et al. [25]

The authors of this study have revealed a scheme to detect severe pharyngitis utilising a deep learning model with smartphone-based self-captured throat images. The authors also claimed that a convolutional neural network (CNN) framework had been observed in the automated detection of severe pharyngitis. The authors have classified the dataset into two interconnected parts; one is affected throat images with pharyngitis; another is typical throat images. Before training the model, the authors adopted a cycle consistency generative adversarial network (CycleGAN) due to the augmentation of the dataset. ResNet50, Inception V-3, MobileNet-v2 models were refined and interpreted to evaluate the severe pharyngitis detection accuracy. The study of the paper has experienced several types of drawbacks. First, the scheme was trained with a small dataset set; 131 for affected throat images and 208 typical images, though they had performed augmentation on the dataset. Second, the authors did not provide a complete guideline of the utilisation of smartphones during self-capturing. Finally, the authors did not give a real-life application of the proposed system. As the authors had claimed, the proposed scheme will be effective on COVID-19 patients during a recent pandemic.

This study presents the summary of each article that has worked on COVID-19 diagnosis utilising other images. Table 4 shows the corresponding table for the short overview of the models. This table presents the adopted models, dataset size, accuracy and contribution of the papers reviewed in this section.

**Table 4:** Summary of each article that worked with other images on COVID-19 diagnosis

References	Model	Data size	Accuracy (%)	Contribution
[51]	CNN	2905	94.6	Proposed a 14-layer convolutional neural network with a notified spatial pyramid pooling module to detect COVID-19 accurately
[52]	AD3D-MIL	899	99.0	Proposed an attention-based deep 3D multiple instance learning (AD3D-MIL)
[53]	GRU-AT, BiLSTM-AT	NA	83.7	Proposed a portable non-contact approach to screen the condition of a patient wearing a mask by analysing the respiratory characteristics from RGB-infrared sensors
[29]	nCOVnet	337	97.0	Proposed a deep learning neural network-based method named nCOVnet, which is used to detect COVID-19 from X-ray images
[54]	DADLMs	344	91.0	Proposed two data-augmentation models to enhance learnability of CNN and ConvLSTM-based deep learning models (DADLMs)
[25]	ResNet18	585	98.0	Proposed deep learning-based decision tree classifications which was a different approach in this COVID-19 classification work

**Fig. 10:**
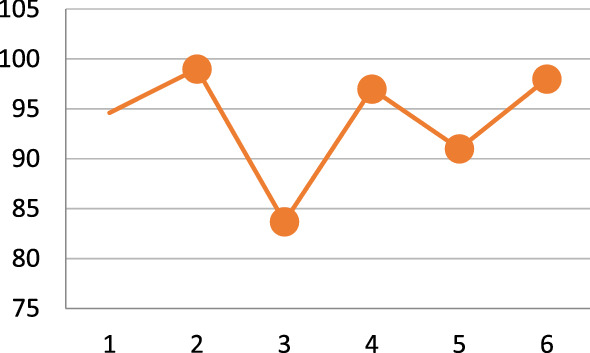
Variation in highest accuracy of the models

From Fig. 10, it can be seen that, except for the GRU-AT and BiLSTM-AT [50], most of the models achieve an accuracy of over 90%. ResNet18 [22], nCOVnet [26], CNN [48], and DADLMs [51] came in second, third, fourth, and fifth place, respectively, with the highest result of 99.0% accuracy achieved by the AD3D-MIL [49] model. Although the dataset used in this proposed work contains less than 1000 data records, the model is capable of maintaining efficiency with less data.

**Fig. 11:**
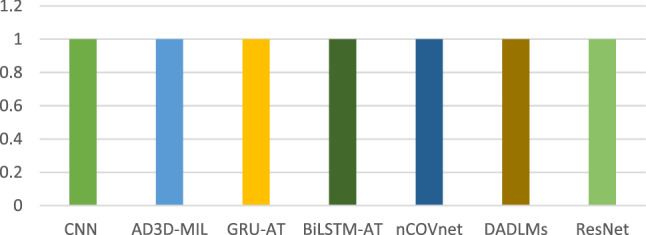
The occurrence of models that worked on other images

From Fig. 11, we can see that no model was used more than once in this insight section, but the main focus point is that most of the algorithms are a modified version of CNN, and these are hyper-tuned for different kinds of approaches.

#### Some Recent Articles on COVID-19 Screening Using Deep Learning

##### Sara et al. [55]

The authors had adopted the conventional deep learning models to find the optimum results in COVID-19 abnormality detection from chest and X-ray images. Though the model achieved the highest accuracy of 99% with DenseNet with Bagging tree classifier, the dataset was very small to work with such a critical topic. To optimise the dataset, image augmentation and the fine-tuning of the weights can be possible solutions to overcome this issue.

##### Samira et al. [56]

The authors of this paper have built an effective mechanism of COVID-19 diagnosis where the authors had utilised WSN models and deep learning. But the paper is very poorly written. They are no obvious models for COVID-19 diagnosis except ANN. The authors’ need a tremendous afford to enrich the methodology.

##### Mohammad et al. [57]

Although new findings imply that CXR images may not be the optimal modality for early COVID-19 identification, other study findings (published in literature studies) corroborate that radiology images reveal important information regarding COVID-19 infection as the disease progresses. As a result, this study can confidently assert that the proposed fusion model is in no way a substitute for a human radiologist. Instead, they anticipate that the current findings will significantly contribute to the expanding acceptance and use of AI-assisted applications in healthcare contexts.

##### Dan et al. [58]

Although this work did not completely investigate the approaches for improving robustness and preventing overfitting, it may serve as a baseline for future model generalisation studies that employ medical data to deploy COVID-19-related classification models in clinical settings. Regarding the identification of COVID-19-positive patients by their medical data, this research will continue to study the boundaries of model generalisation concerning enhancing the algorithm and the intra- and inter-source data variability. Overall, the deep learning models performed well on the unseen test set from the same distribution that they were trained on, indicating no typical overfitting problem with the training data in this study.

##### DandiYang et al. [59]

One of the paper’s key conclusions is that by combining more public databases, data fusion models might improve diagnostic and predictive performance even further. The other is that the concentrated models could effectively support virologists in diagnosing COVID-19 and radiologists in the fight against COVID-19 outbreaks, arriving at vital patient diagnoses in minutes, which could be critical in their treatment.

##### Arpita et al. [55]

The proposed design greatly improves the model’s diagnostic ability and obtains a high AUC score. As a result, KarNet has been shown to outperform the present state of the art in accurately classifying COVID-19 patients. The proposed approach can improve COVID-19 testing methods because most hospitals have CT scanners. As a result, this model can be used as an automated alternative testing method, potentially saving them time and lives of infected patients before it is too late.

##### Subrato et al. [60]

The proposed CO-ResNet method is an optimised version of ResNet101 that uses hyperparameter adjustment to accomplish optimisation. It outperformed other typical ResNet models in terms of results. COVID-19 has a detection rate of 98.74% in ResNet101 assessment data.

##### Julia et al. [61]

Deep learning techniques for lung ultrasound imaging computer-assisted interpretation offer a viable path forward for COVID-19 screening and diagnosis. We discovered that the InceptionV3 network, in particular, gives the most promising predictive outcomes among all AI-based techniques studied in this paper. Based on ultrasound imaging, InceptionV3 and Xception-based models can be used further to build a viable computer-assisted screening method for COVID-19.

This study further depicts the summary of recent articles that are worked on the COVID-19 diagnosis utilising a variety of images. Table 5 shows the corresponding table for the short overview of the models. This table presents the adopted models, dataset size, accuracy and contribution of the papers reviewed in this section.

**Table 5:** Summary of recent articles that worked with various types of images on COVID-19 diagnosis

References	Model	Data size	Accuracy (%)	Contribution
[55]	DenseNet	137	99.0	The utilisation of different types of conventional deep learning algorithms
[56]	ANN	N/A	94.0	Proposed an attention-based deep 3D multiple instance learning (AD3D-MIL)
[57]	ANN	N/A	94.0	A new convolutional neural network (CNN)-based deep learning fusion framework was proposed in this study, which uses the transfer learning concept to combine parameters (weights) from different models into a single model to extract features from images, which are then fed to a custom classifier for prediction
[58]	DNN	N/A	97.0	This work aims to assess the severity of the problem by comparing deep learning (DL) classification models trained to detect COVID-19-positive patients using 3D computed tomography (CT) datasets from various nations
[35]	VGG16, DenseNet121, ResNet50, and ResNet152	8461	99.0	This study used four powerful pre‑trained CNN models, VGG16, DenseNet121, ResNet50, and ResNet152, for the COVID‑19 CT‑scan binary classification challenge. The suggested Fast.AI ResNet framework was meant to find the appropriate architecture, preprocessing automatically, and training parameters for the models
[60]	DenseNet201, VGG16, ResNet50V2, and MobileNet	2481	97.0	The proposed methodology uses transfer-learning pre-trained models to classify COVID-19 (positive) and COVID-19 (negative) patients. They describe the creation of KarNet, a deep learning framework that uses pre-trained models (DenseNet201, VGG16, ResNet50V2, and MobileNet) as its backbone
[61]	ResNet152	5935	92.08	The suggested CO-ResNet is created by tweaking the hyperparameters of the standard ResNet 101. CO-ResNet is used to analyse a new dataset of 5935 X-ray pictures culled from two publicly available sources. Their CO-ResNet was optimised for identifying COVID-19 vs pneumonia with normal healthy lung controls by using resizing, augmentation, and normalisation, as well as testing different epochs
[28]	VGG19, InceptionV3, Xception, and ResNet50	261	89.1	They used VGG19, InceptionV3, Xception, and ResNet50 to modify several pre-trained deep learning architectures. They used the publicly available POCUS dataset for training and fine-tuning, which contains 3326 lung ultrasound frames of healthy, COVID-19, and pneumonia patients

**Fig. 12:**
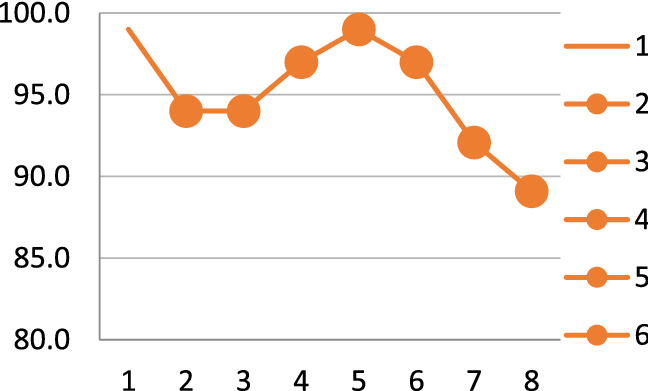
Variation in highest accuracy of the models

Figure 12 shows the maximum accuracy in the previous paper over the various sophisticated deep learning techniques. From this figure, it can be delineated that the highest accuracy was recorded to be 99.0% among the different state-of-the-art methods. It is also noticeable that 97% accuracy appeared in the previous studies that have been taken into consideration in the second-largest model after the 99% accuracy. In contrast, 84% accuracy was the lowest in the existing studies.

**Fig. 13:**
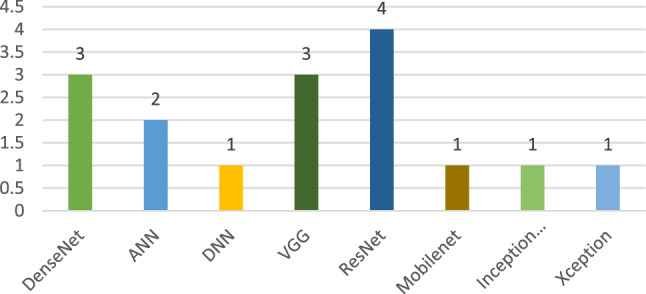
The occurrence of models in recent articles

In addition to the maximum accuracy, this research also concentrated on extracting meaningful insights by analysing the performance based on the individual models. Considering Fig. 12, it is observed that the authors of the current study have carried out their research and experimented through various state-of-the-art deep learning techniques to illustrate, DenseNet, ANN, DNN, VGG, ResNet, MobileNet, InceptionV3 and Xception. These algorithms have benchmark performance on top of the multimodal medical imaging modalities. The ResNet model was the most frequent model among the various algorithms utilised previously. The ResNet model was trained through the ImageNet dataset using thousands of images to distinguish objects easily. Hence, it gives a good accuracy while classifying images. Besides, the DenseNet and VGG models were significant after the ResNet model. Even though the Mobilenet, InceptionV3 and Xception models are found to be satisfactory for classification related tasks, still, for the case of detecting COVID-19 disease, these models have not provided an adequate performance that can be observed explicitly by looking at Fig. 13.

## Summary of COVID-19 Diagnosis Using Deep Learning

This research has summarised some of the developed systems for COVID-19 diagnosis, illustrated in Fig. 6. The review has involved analysing data sources, the number of images and methodology proposed, the methods of data partitioning, the diagnostic deep learning technique used, and finally, the accuracy graph to calculate the accuracy of the previous analysis. Some of the systems used many images, but the number of samples for COVID-19 cases is comparatively limited. Throughout the research, both the multiclass and image augmentation methods are considered. Some of the systems used cross-validation methods concerning data partitioning, and others used the approach of hold-out. We have made comparisons and observations based on the contribution of the past models.

It is evident from the results that most of the developed systems used a transfer-learning approach, 2D DL framework, attention mechanism [1]. However, there are generalizability and overfitting issues that can be found in the case of 2D DL framework. In another case, [40] combined CNN and multi-objective differential evolution (MODE) function to detect COVID-19 patients. Parameter tuning is required to ensure more optimal performance, as we have observed while reviewing. The study [1] proposed DenseNet201 based on deep transfer learning methods for classifying COVID-19 patients. We found that in the case of medical imaging, transfer learning shows minimal performance achievement. Another study [54] shows machine learning and deep learning models for efficient data-augmented detection of COVID-19. However, no significant observations were made in this study. Based on the dataset, the performance of the developed systems varied. As almost all the procedures used various data sizes for the experiment, the performance of the reviewed systems is not comparable. In terms of comparison of image modalities, the approach based on CT performed relatively better than the method based on X-rays.

It is predicted that the study that has been conducted using CT samples would be applicable for real-time testing. The X-ray-based proposed systems require real-time testing with target individuals before application. Table 6 summarises accuracy findings from the literature review and sensitivity, Specificity, F1-score and AUC.

**Table 6:** Summary of accuracy findings from literature review along with sensitivity, specificity, F1-score and AUC

References	Accuracy	Sensitivity	Specificity	F1-score	AUC
3D ResNet34 [49]	87.5%	86.9%	90.1%	82.0%	94.4%
DL [46]	85%	79.35%	71.43%	90.11%	N/A
ResNet18 [25] 98%	99%	97%	N/A	98%	
CapsuleNetwork [50]	97.24%	84.22%	91.79%	84.21%	N/A
SqueezeNet [24]	99.24%	98.86%	100%	99.43%	N/A
VGG16 [21]	88.10%	97.62%	78.57%	N/A	N/A
DarkNet [21]	98.08%	95.13%	95.3%	96.51%	N/A
SqueezeNet [32]	100%	N/A	99.67%	99.67%	N/A
AI system [52]	92.49%	94.93%	91.13%	N/A	97.97%
CNN [40]	Improves by 1.97%	Improves by 1.82%	Improves by 1.68%	Improves by 2.09%	N/A
CycleGAN [25]	95.3%	92.9%	96.8%	N/A	99%
ResNet18 [21]	86.7%	N/A	N/A	N/A	N/A
CNN [36]	99.01%	N/A	N/A	N/A	99.72%
Inception Resnet V2 [27]	92.18%	92.11%	96.06%	92.07%	N/A
DL [43]	90.8%	84%	93%	N/A	N/A
ResNet50 [47]	99.87%	99.58%	100.00%	N/A	N/A
[50]	N/A	96%	N/A	86%	86%
DL [41]	97.4%	N/A	94.7%	97.1%	96.9%
CNN [31]	93.2%	96.1%	N/A	N/A	N/A
CoroNet [38]	99%	N/A	98.6%	98.5%	N/A
SVM [37]	98.97%	89.39%	99.75%	96.72%	N/A
DL [50]	92.2%	86.4%	93.3%	91.5%	N/A
DL[28]	96%	96%	98%	94%	N/A
DenseNet201 [1]	99.82%	N/A	99.23%	99.82%	N/A
RGB [53]	83.69%	90.23%	76.31%	N/A	N/A
Instance Learning [52]	97.9,%	N/A	N/A	N/A	99.0%
Inf-Net [44]	N/A	87%	97.7%	N/A	N/A
ResNet18 [22]	88.89%	N/A	96.4%	84.4%	N/A
CNN-LSTM [23]	99.4%	99.3%	99.2%	98.9%	99.9%
VGG and CNN based [26]	73%	N/A	N/A	N/A	N/A
DL [51]	94.6%	N/A	N/A	N/A	N/A
ResNet [34]	89%	N/A	N/A	N/A	N/A
Curvelet [35]	99.69%	N/A	96.05%	92.91%	N/A
[54]	99%	N/A	N/A	N/A	N/A

## Conclusion

The standard and most-used method for detecting COVID-19 is RT-PCR. Still, many scientists and researchers claim that it may suffer from limitations and shortcomings that hinder them from implementation in a real-life scenario. Therefore, we have to find an alternative and effective diagnosis and screening method. Furthermore, there are other concerns regarding model training and transfer learning. Since the datasets are not mature and readily available, it is hard for the researcher and scientist to develop specific models. As pre-trained models are trained on specific datasets, the dataset of COVID-19-infected patients may differ from those. Therefore, more experiments are needed to improve deep learning techniques. We have compared most of the well-known models here and have identified the efficiency of the various models by describing some of the unique processing techniques, which is extremely important in the field of medical image analysis. These findings will help us and future researchers to pursue any of the frameworks and contribute to improve these models.

Confirmed COVID-19 cases increased daily throughout 2020, with millions of deaths, while high levels of mortality continued in 2021. Early diagnosis and screening of COVID-19 patients may limit the spreading of this highly contagious disease. Early detection DL-based imaging shows various limitations, and many researchers have concerns about these methods. From our observations, we have to identify the limitations to get satisfactory results for COVID-19. We have to classify pneumonia and other respiratory lung disease patients first; then use these datasets for the classification and detection of COVID-19 patients. This paper addresses those issues and also demonstrates the types of models.

## Future Scope

This study has aimed to point to the gaps in the developed systems, which will help future researchers to understand COVID-19-related DL models and consider the shortcoming to build a better system. In the future, we will extend this research by investigating the results with a meta-analysis. Second, we want to compare the algorithms of the qualified papers of this research. The comparison will help us and future researchers to enhance the quality of research on machine learning and deep learning and serve as a benchmark in the research community. Finally, we will extend this research to investigate anatomical and organ models to construct our own patient-specific model from medical images. Adaptable image analysis techniques will enable efficient development to recognise any disease like COVID-19.
